# 

**Published:** 1896-12

**Authors:** 


### TABLE OF CONTENTS

ORIGINAL COMMUNICATIONS. PAGE

The Röntgen Ray. By WILLIAM LINCOLN SMITH . . . . . . . . . . . 765

Pyorrhœa Alveolaris from a New Stand-Point. By WILLAM H. TRUEMAN, D.D.S., Philadelphia . . . . . . . . . . . . . . . . . . . . . . . . 774

Skeleton Notes on Cataphoresis. By DR. LOUIS JACK . . . . . . . . . . 783

ABSTRACTS AND TRANSLATIONS.

Putting a Porcelain Facing on a Living Honey-Combed Incisor. By P. HEADRIDGE, L.D.S.I. . . . . . . . . . . . . . . . . . . . . . . 787

REPORTS OF SOCIETY MEETINGS.

American Dental Association . . . . . . . . . . . . . . . . . . . . . 788

American Academy of Dental Science . . . . . . . . . . . . . . . . . 806

Academy of Stomatology . . . . . . . . . . . . . . . . . . . . . . . 821

EDITORIAL.

December Thoughts . . . . . . . . . . . . . . . . . . . . . . . . . 828

The Separation of the National Associations . . . . . . . . . . . . . . 830

BIBLIOGRAPHY . . . . . . . . . . . . . . . . . . . . . . . . . . . 832

OBITUARY.

Resolution of Respect to the Memory of Dr. E. A. Stebbins . . . . . . . 834

Dr. E. L. Childs . . . . . . . . . . . . . . . . . . . . . . . . . . 835

CURRENT NEWS.

Northeastern Dental Association . . . . . . . . . . . . . . . . . . . 835

New Jersey State Dental Society . . . . . . . . . . . . . . . . . . . 836

